# Abdominal Obesity, Race and Chronic Kidney Disease in Young Adults: Results from NHANES 1999-2010

**DOI:** 10.1371/journal.pone.0153588

**Published:** 2016-05-25

**Authors:** Harini Sarathy, Gabriela Henriquez, Matthew K. Abramowitz, Holly Kramer, Sylvia E. Rosas, Tanya Johns, Juhi Kumar, Amy Skversky, Frederick Kaskel, Michal L. Melamed

**Affiliations:** 1 Department of Medicine, Albert Einstein College of Medicine / Montefiore Medical Center, Bronx, New York, United States of America; 2 Department of Pediatrics, Albert Einstein College of Medicine / Montefiore Medical Center, Bronx, New York, United States of America; 3 Department of Epidemiology and Population Health, Albert Einstein College of Medicine, Bronx, New York, United States of America; 4 Departments of Public Health Sciences and Medicine, Division of Nephrology and Hypertension, Loyola University Health Sciences Center, Maywood, Illinois, United States of America; 5 Joslin Diabetes Center and Beth Israel Deaconess Medical Center, Boston, Massachusetts, United States of America; 6 Department of Pediatrics, Weill Cornell Medical Center, New York, New York, United States of America; Innsbruck Medical University, AUSTRIA

## Abstract

**Objective:**

Kidney dysfunction in obesity may be independent of and may precede the development of hypertension and/or diabetes mellitus. We aimed to examine if abdominal obesity is associated with early markers of CKD in a young healthy population and whether these associations differ by race and/or ethnicity.

**Methods:**

We analyzed data from the NHANES 1999–2010 for 6918 young adults ages 20–40 years. Abdominal obesity was defined by gender criteria of waist circumference. CKD markers included estimated glomerular filtration rate and albuminuria ≥30 mg/g. Race stratified analyses were done overall and in subgroups with normal blood pressures, normoglycemia and normal insulin sensitivity. Awareness of CKD was assessed in participants with albuminuria.

**Results:**

Abdominal obesity was present in over one-third of all young adults and was more prevalent among non-Hispanic blacks (45.4%) versus Mexican-Americans (40.6%) or non-Hispanic whites (37.4%) (P-value = 0.004). Mexican-American young adults with abdominal obesity had a higher odds of albuminuria even among those with normal blood pressure, normal glucose, and normal insulin sensitivity [adjusted odds ratio 4.5; 95% confidence interval (1.6–12.2), p = 0.004]. Less than 5% of young adults with albuminuria of all races and ethnicities had been told they had kidney disease.

**Conclusion:**

Abdominal obesity in young adults, especially in Mexican-Americans, is independently associated with albuminuria even with normal blood pressures, normoglycemia and normal insulin levels. Greater awareness of CKD is needed to protect this young population from long-standing exposure to abdominal obesity and early progressive renal disease.

## Introduction

The obesity epidemic affects more than a third of adults (body mass index (BMI) ≥30 kg/m^2^) in the United States, according to data from the National Health and Nutrition Examination Survey (NHANES) 2011–2012 survey cycle[1]. Obesity is closely linked to the development of hypertension, dyslipidemia and diabetes mellitus that together constitute the metabolic syndrome. Together, they are well established independent risk factors for cardiovascular disease (CVD) and chronic kidney disease (CKD) [2]. Obesity is associated with incident and prevalent microalbuminuria and higher glomerular filtration rate (GFR), or hyperfiltration, which together with albuminuria may herald progressive kidney dysfunction and glomerulosclerosis [2–4]. Moreover, microalbuminuria is considered a non-traditional independent risk factor for CVD in adults [5–7].

Emerging evidence suggests that kidney dysfunction in obesity may be independent of traditional CKD risk factors namely hypertension and diabetes mellitus, and may be apparent long before these factors develop in patients with the metabolic syndrome [8, 9]. The CARDIA Study in healthy white and black young adults showed a rapid decline in estimated GFR (eGFR) with higher BMI [10]. However, they did not assess albuminuria or include Hispanics, now the largest racial/ ethnic minority in the US. The young adult population is at a high risk of obesity-related future kidney disease and provides opportunities for preventive interventions [11].

Waist circumference appears to be a better marker of obesity-related health risks than other surrogates for obesity [3, 12]. In the era of personalized medicine, evidence that abdominal obesity has different associations with CKD risk factors in different racial/ethnic groups can help clinicians to tailor screening and therapy and can help guide research to evaluate underlying biologic differences. We aimed to estimate the association of abdominal obesity (determined by waist circumference) with CKD risk factors and early CKD markers, in young adults ages 20–40 years and to assess differences in these associations between Mexican-Americans and non-Hispanic whites and blacks using nationally representative NHANES data.

## Materials and Methods

NHANES is a continuous cross-sectional, multi-stage stratified, clustered probability sample survey performed in 2 year cycles of participants that are nationally representative of the US non-institutionalized civilian population. We combined data from 6 cycles of NHANES (from 1999–2000 to 2009–10) in accordance with NCHS recommendations [13]. Details of the survey design were similar across all NHANES and are described elsewhere [14–16]. All participants gave written informed consent and underwent standardized home interviews, as well as physical examination and laboratory testing at a mobile health center. NHANES was approved by the NCHS Research Ethics Review Board. The institutional review board at the Albert Einstein College of Medicine exempted this analysis.

We analyzed data from 6918 non-pregnant young adults ages 20–40 years over the 12 year period from 1999–2010 self identifying as non-Hispanic white, non-Hispanic black and Mexican-American. These participants, representing nearly 54.3 million people, had complete information on variables of interest. We excluded 20571 adults over 40 years age, 29670 participants younger than 20 years age, 595 persons from other races, and those with missing values on the following variables (n = missing): waist circumference (n = 942), weight (n = 11), height (n = 3), serum creatinine (n = 2327), urine creatinine (n = 47), systolic blood pressure (n = 255), hemoglobin A1c (HbA1c) (n = 16), total cholesterol (n = 5), C-Reactive Protein (CRP) levels (n = 2), poverty income ratio (PIR) (n = 439), smoking status (n = 3), and kidney disease awareness (n = 6). Fasting serum samples were obtained from a subsample of randomly selected individuals, thus further excluding participants with missing values of triglycerides (n = 3593), LDL-cholesterol (n = 150), glucose (n = 3), insulin (n = 13); leading to 3159 young adults for the fasting analyses. Non-Hispanic blacks, Mexican-Americans, and low-income persons were oversampled in all cycles to obtain more accurate estimates.

### Study variables

#### Interview

Self-reported variables included age, sex, race/ethnicity, smoking status and physical activity. Race/ethnicity was categorized as non-Hispanic white, non-Hispanic black and Mexican-American. The poverty income ratio (PIR) is calculated by dividing family income over a poverty threshold for that year as defined by the U.S. Census. We categorized PIR≤1 as below the poverty threshold, 1>PIR ≤2 as being at or above poverty level, and PIR >2 as >200% above poverty level. Smoking status was categorized as never, former and current smokers based on responses to: “Have you smoked at least 100 cigarettes in your life?” and “Do you now smoke cigarettes?” Physical activity was based on the participant’s metabolic expenditures in the past month, and categorized as low physical activity intensity (<3 METS), moderate (3–6 METS), or vigorous intensity (>6 METS), as suggested by NHANES/NCHS [17].

#### Physical Examination

Anthropometric data including height, weight, and waist circumference were measured during the physical examination. Waist circumference was measured just above the uppermost lateral border of the right ilium, at the end of normal expiration in standing position, to the nearest 0.1 cm with the tape snug but not compressing the skin [18]. For this study, abdominal obesity was the main predictor of interest and was defined as waist circumference ≥102 cm in males and ≥88 cm in females by ATP-III criteria [19]. BMI was calculated by dividing the weight (in kilograms) by the square of the height (in meters) and categorized as normal (<25 kg/m^2^), overweight (≥25 and≤30 kg/m^2^) and obese (>30 kg/m^2^).

Blood pressure readings were recorded on the same day on the right arm with the participant in a seated position. A minimum 30 second interval occurred between readings during which time the manometry tubing was fully disconnected [20]. The initial reading was taken after the participant was rested for 5 minutes, and a fourth reading was taken if the any of the three previous readings were interrupted or incomplete [20]. Hypertension was defined as average SBP >140 mmHg or average DBP >90 mmHg or if they had been told twice or more that they had high blood pressure or were prescribed anti-hypertensive medications (coded as positive based on the answer to “Because of your high blood pressure, have you ever been told to take a prescribed medicine?”). We did not assess the class of antihypertensive medication prescribed.

#### Laboratory Analyses

Laboratory samples were processed in accordance with a standardized protocol. For this analysis, elevated CRP was defined as ≥0.21 mg/dl. Fasting blood samples were taken from randomly selected participants in the morning sessions with assured fasting status ≥8 hours. Appropriate corrections were made for the assay variability in measurements of lipid fractions, glucose, and insulin levels across the survey cycles [21–25]. LDL was calculated using the Friedewald equation: LDL(mg/dl) [TC(mg/dl)–HDL(mg/dl)–(TG/5) (mg/dl)]. Impaired fasting glucose was defined as a fasting glucose level between 100 and 125 mg/dl without a history of diabetes mellitus or HbA1c ≥5.7 and <6.5%. Diabetes mellitus was defined as fasting glucose level >125 mg/dl or HbA1c ≥6.5%. We calculated the Homeostatic Model Assessment for Insulin Resistance values [HOMA-IR = fasting serum insulin (mU/L) x fasting plasma glucose (mg/dl)]/405].

Serum creatinine was measured using the Jaffe method in all participants and was used with recalibrations as recommended [26]. The CKD-EPI equation based on age, sex, race and standardized serum creatinine values, was used to calculate eGFR [27].

Random morning urine samples were collected during the examination and frozen non-hematuric samples were analyzed in the same laboratory during all surveys for urine albumin using fluorescent immunoassay and urine creatinine by the Jaffe method. Urinary albumin/creatinine ratio (UACR) was expressed as milligrams of albumin per gram of creatinine (mg/g) and albuminuria was defined as UACR ≥30 mg/g to represent moderately increased and clinically significant levels, as per KDIGO guidelines.[28] KDIGO recommended cutoffs for sex-specific criteria for albuminuria were also used and defined as UACR ≥17 mg/g in males, and ≥25 mg/g in females.[29] To elucidate the distribution of albuminuria, we categorized it as UACR<30 mg/g (normal), 30≥UACR<100 mg/g, 100≥UACR<300 mg/g and UACR≥300 mg/g.

### Statistical analyses

The analysis accounted for the cluster and strata design of the complex survey and the sampling weights to adjust for the unequal probabilities of selection, oversampling, and non-response. Weighted analyses were performed using the ‘survey’ command with Taylor series (linearization) method for standard errors (SE) in Stata 11.0. Estimates and standard errors were weighted for combined survey years and interview or examination-related variables. The weighted estimates were projected for young adults in a standard U.S. population based on the 2000 US Census Counts.[13] A p-value of less than 0.05 in two-sided hypothesis testing was the level of statistical significance for all analyses. We did not adjust for multiple comparisons, given the hypothesis-generating nature of the analyses.[30]

#### Stratified analyses

Participants were stratified by race/ethnicity as non-Hispanic white, non-Hispanic black or Mexican-American. Participant characteristics/covariates were examined as weighted sample means and proportions and tested for significant differences with Student’s t-test for continuous variables and Pearson chi-square (with Rao-Scott correction F-statistic) tests for categorical variables. We compared participant characteristics by race for participants with and without missing data. We also tested for interactions between abdominal obesity and CKD risk variables by race/ethnicity to evaluate whether there were substantial differences by race/ethnicity.

#### Hypothesis testing

We analyzed the association of our main exposure variable, abdominal obesity, with CKD markers (albuminuria and eGFR) in multivariable logistic and linear regression analyses adjusted for age, sex, race/ethnicity, PIR, smoking status, and survey year, as well as CKD risk variables including systolic and diastolic blood pressures, HbA1c, total cholesterol, HDL-cholesterol and elevated CRP levels. Weighted analyses were performed on subsamples with non-missing values of variables. Variables requiring fasting blood samples were analyzed separately with corresponding weights. Adjusted models did not include covariates requiring fasting status or physical activity, as sample sizes would be severely compromised. To assess an independent association of abdominal obesity with CKD markers, we created sub-groups of young adults with non-hypertensive blood pressures (<140/90 mmHg), normoglycemia (fasting blood glucose <100 mg/dl or HbA1C<5.7%) and normal insulin sensitivity (HOMA-IR <75^th^ percentile) [31].

#### Kidney disease awareness

Kidney disease awareness among young adults with albuminuria was measured by weighted proportions of positive answers to the question: “Ever told you had weak/failing kidneys?” Race-stratified analyses were performed in sub-samples with non-missing values of age, race/ethnicity, abdominal obesity, response to the awareness question, and albuminuria. Small sample sizes precluded further analyses.

#### Sensitivity analysis

We performed sensitivity analysis to assess whether adjusting for physical activity altered the association of abdominal obesity with CKD risk factors and CKD markers. Additionally, we redefined normal blood pressures to be ≤120/80mmHg and repeated the analyses in individuals with normotension, normoglycemia and normal insulin sensitivity.

## Results

### Comparison of participants with and without missing variables

We examined the distribution of CKD risk factors in NHANES participants with missing data on one or more variables of interest. Among 1153 participants with missing data belonging to one of the three races/ethnicities of interest, there was a greater proportion of non-Hispanic blacks (18.8%) and Mexican Americans (20.6%) compared to participants with complete data (12.1% and 18.5% respectively). The distribution of CKD risk factors was similar to those with complete data but with more abnormal values among participants with missing data (S1 Table and Table 1). Notably, prevalence of elevated CRP and albuminuria were higher in the missing data group though eGFR was similar in distribution.

**Table 1:** Weighted baseline characteristics of CKD risk factors and CKD markers in young adults ages 20–40 years by race^¥^.

	Non-Hispanic Whites (N = 3226) (37,606,080.5)			Non-Hispanic Blacks (N = 1440) (6,579,829.4)			Mexican-Americans (N = 2252) (10,111,528.9)		
	No abdominal obesity 1897(62.8%)	Abdominal Obesity 1329 (37.2%)	P	No abdominal obesity 781 (54.8%)	Abdominal Obesity 659 (45.2%)	p	No abdominal obesity 1244(59.6%)	Abdominal Obesity 1008 (40.4%)	P
**Age**(yrs)[Mean(SD)]	29.9 (0.2)	31.7 (0.2)	<0.001	29.2 (0.3)	30.4 (0.3)	0.001	29.4 (0.3)	30.8 (0.2)	<0.001
**Gender** [n (%)]									
Males	1030 (55.3)	459 (40.6)	<0.001	489 (60.9)	182 (26.2)	<0.001	759 (64.6)	281 (34.8)	<0.001
Females	867 (44.7)	870 (59.4)		292 (39.1)	477 (73.8)		485 (35.4)	727 (65.2)	
**Per-capita Income Ratio (PIR)** [n (%)]									
Below poverty	259 (11.5)	218 (12.6)	0.37	205 (27.0)	189 (28.3)	0.39	377 (27.7)	341 (32.2)	0.25
Above poverty	367 (17.8)	259 (19.4)		195 (25.0)	180 (27.7)		442 (35.8)	341 (33.2)	
200% above poverty	1271 (70.7)	852 (68.1)		381 (48.0)	290 (44.0)		425 (36.5)	326 (34.6)	
**Smoking Status** [n (%)]									
Never	939 (50.9)	663 (49.9)	0.03	519 (67.1)	466 (71.7)	0.003	780 (61.4)	700 (65.5)	0.04
Former	280 (14.8)	264 (18.6)		399 (4.8)	55 (7.1)		169 (13.7)	149 (14.8)	
Current	678 (34.3)	402 (31.5)		223(28.2)	138 (21.2)		295 (24.9)	159 (19.7)	
**BMI category** (kg/m^2^) [n (%)]									
Normal (<25)	1304 (67.3)	79 (3.6)	<0.001	441 (56.8)	18 (1.9)	<0.001	653 (50.5)	32 (2.1)	<0.001
Overweight (25–29)	563 (30.9)	431 (30.2)		285 (36.5)	117 (17.6)		532 (44.4)	316 (28.7)	
Obese (≥30)	30 (1.7)	819 (66.2)		55 (6.8)	524 (80.4)		59(5.1)	660 (69.2)	
**BMI** (kg/m^2^) [Mean(SD)]	23. 7 (0.1)	32.9 (0.2)	<0.001	24.4 (0.1)	35.4 (0.3)	<0.001	24.8 (0.1)	33.0 (0.2)	<0.001
**Systolic BP** (mmHg)Mean(SD)]	112.9 (0.3)	116.7 (0.5)	<0.001	117.0 (0.5)	119.0 (0.6)	0.004	113.9 (0.4)	115.3 (0.5)	0.03
**Diastolic BP** (mmHg)[Mean(SD)]	68.6 (0.3)	71.7 (0.5)	<0.001	69.8 (0.5)	70.6 (0.5)	0.22	66.8 (0.4)	69.1 (0.5)	<0.001
**Hypertension** [n (%)]	136 (7.1)	231 (18.1)	<0.001	84 (10.7)	156 (24.2)	<0.001	72 (6.6)	120 (13.3)	0.001
**HbA1C** (%)[Mean(SD)]	5.1 (0.01)	5.3 (0.03)	<0.001	5.2 (0.02)	5.5 (0.04)	<0.001	5.2 (0.02)	5.4 (0.04)	<0.001
**Total Cholesterol** (mg/dl) [Mean(SD)]	185.6 (0.9)	200.5 (1.2)	<0.001	179.9 (1.4)	191.5 (1.9)	<0.001	187.6 (1.2)	193.9 (1.6)	0.001
**HDL-Cholesterol** (mg/dl) [Mean(SD)]	53.8 (0.4)	46.7 (0.4)	<0.001	56.6 (0.6)	52.3 (0.7)	<0.001	50.4 (0.4)	47.2 (0.5)	<0.001
**Elevated CRP** (>21mg/dl) [n (%)]	499 (24.2)	879 (62.4)	<0.001	196 (24.3)	472 (70.6)	<0.001	375 (29.4)	731 (68.6)	<0.001
**Markers of kidney damage**									
**eGFR** [Mean (SD)] (ml/min/1.73m^2^)	104.8 (0.5)	106.1 (0.5)	0.09	114.7 (1.0)	119.5 (0.8)	<0.001	113.5 (0.6)	114.9 (0.6)	0.04
**Albuminuria** (mg/g) [n(%)]	88 (4.5)	77 (5.6)	0.17	37 (4.7)	46 (6.6)	0.09	48 (3.6)	101 (11.6)	<0.001
**Sex-specific albuminuria** (mg/g) [n (%)]	144 (7.1)	106 (7.9)	0.43	62 (8.1)	66 (9.5)	0.26	84 (6.4)	131 (14.4)	<0.001

Fasting Sample Analysis	Non-Hispanic Whites (N = 1452) (38,957,077.4)			Non-Hispanic Blacks (N = 708) (7,351,235.5)			Mexican-Americans (N = 999) (10,353,432.3)		
	No abdominal obesity 857 (63.2)	Abdominal Obesity 595 (36.8)	P	No abdominal obesity 386 (53.6)	Abdominal Obesity 322 (46.3)	p	No abdominal obesity 536 (56.8)	Abdominal Obesity 463 (43.2)	p
**Triglycerides** (mg/dl) [Mean(SD)]	103.0 (2.1)	145.4 (2.8)	<0.001	84.0 (2.9)	106.7 (4.5)	<0.001	114.9 (3.6)	144.3 (4.3)	<0.001
**LDL**(mg/dl) [Mean(SD)]	108.9 (1.4)	119.2 (1.5)	<0.001	104.8 (2.1)	115.7 (2.2)	0.001	112.3 (1.6)	115.5 (2.1)	0.23
**Glucose** (mg/dl) [Mean(SD)]	93.2 (0.4)	99.1 (1.0)	<0.001	92.1 (0.5)	100.6 (2.2)	<0.001	98.0 (0.9)	99.6 (2.5)	0.57
**Insulin** (mg/dl) [Mean(SD)]	5.7 (0.1)	14.4 (0.7)	<0.001	7.3 (0.3)	16.8 (0.7)	<0.001	8.5 (0.7)	14.8 (0.6)	<0.001
**HOMA** [Mean (SD)]	1.9 (0.03)	3.1 (0.1)	<0.001	2.2 (0.1)	3.3 (0.1)	<0.001	2.4 (0.05)	3.3 (0.1)	<0.001
**Diabetes status [n (%)]**									
Non-diabetic	687 (80.8)	425 (66.5)	<0.001	319 (82.4)	222 (69.1)	<0.001	359 (65.4)	318 (68.1)	0.19
Glucose intolerance	165 (18.5)	152 (30.0)		67 (17.6)	86 (26.0)		170 (33.4)	146 (29.0)	
Diabetes	5 (0.7)	18 (3.5)		0 (0)	14 (4.9)		7 (1.2)	9 (2.9)	
**Insulin resistance status [n (%)]**									
Normal	760 (92.8)	324 (55.8)	<0.001	310 (86.5)	135 (43.1)	<0.001	412 (81.9)	231 (52.2)	<0.001
Insulin resistance (HOMA ≥75^th^%tile)	59 (7.2)	237 (44.2)		49 (13.5)	169 (56.9)		98 (18.1)	216 (47.8)	

^¥^ For categorical variables, % are calculated from column-wise proportions.

### Baseline characteristics

The distributions of baseline characteristics in individuals with and without abdominal obesity by race/ ethnicity group are shown in Table 1. Young non-Hispanic blacks were more affected by abdominal obesity (45.2%), than Mexican-Americans (40.4%) and non-Hispanic whites (37.2%) (P-value = 0.004) (Table 1). Across all races, individuals with abdominal obesity were more commonly older, female, obese / overweight by BMI, and never smokers. Additional insight gained from exploratory analysis of categorical variables for row-wise proportions i.e. in each level of the variable, showed that abdominal obesity in all races/ethnicities was higher in former smokers (S2 Table).

CKD risk factors were more commonly abnormal in those with abdominal obesity (Table 1), with blacks showing higher blood pressures and HOMA-derived insulin resistance, but lower lipid levels. CKD markers, albuminuria and eGFR, were also increased in the abdominal obesity category. Across races, Mexican Americans with abdominal obesity had a significantly higher prevalence of albuminuria compared to those without abdominal obesity (11.6% vs. 3.6%, p<0.001) (Table 1). Most of the albuminuria levels were in the 30–100 mg/g range across all races (S3 Table). Overall, eGFR (ml/min/1.73m^2^) was higher in those with abdominal obesity compared to those without abdominal obesity (non-Hispanic blacks: 119.5 vs. 114.7, p<0.001; Mexican-Americans 114.9 vs. 113.5, p = 0.04; non-Hispanic whites (106.1 vs. 104.8, p = 0.09).

### Abdominal obesity and CKD risk factors and early CKD markers

In multivariable analyses, abdominal obesity in young adults remained significantly associated with increased levels and odds of CKD risk factors as described in baseline characteristics. Effect modification by race was significant in the associations of abdominal obesity with hemoglobin A1c (p-interaction = 0.025), total cholesterol (p-interaction = 0.002), HDL cholesterol (p-interaction<0.001), albuminuria (p-interaction<0.001) and HOMA levels (p-interaction = 0.002). It appeared that abdominal obesity was associated with higher hemoglobin A1c and HOMA-IR levels in all racial/ethnic subgroups but more so in non-Hispanic blacks. In contrast, abdominal obesity was associated with higher total cholesterol and lower HDL cholesterol levels more markedly in non-Hispanic whites and blacks compared to Mexican-Americans (Table 2). Mexican-Americans with abdominal obesity had significantly higher odds of albuminuria [odds ratio [OR] 3.0, 95% C.I (1.7–5.4), p = 0.001], [sex-specified albuminuria: 2.1 (1.4–3.2), p<0.001]. Abdominal obesity was only associated with a higher eGFR in non-Hispanic whites [β = 1.9 (SE 0.6), p = 0.005] (Table 2).

**Table 2:** Association between abdominal obesity and CKD risk Factors in White/Black and Mexican-American young adults 20–40 yrs^£^.

	Non-Hispanic Whites (N = 3226) (37,606,080.5)		Non-Hispanic Blacks (N = 1440) (6,579,829.4)		Mexican-Americans (N = 2252) (10,111,528.9)	
Overall	Unadjusted	Adjusted^¶^	Unadjusted	Adjusted^¶^	Unadjusted	Adjusted^¶^
**Systolic BP**	3.8 (0.5)*	4.7 (0.5)*	2.0 (0.7)*	3.1 (0.9)*	1.4 (0.6)*	3.5 (0.6)*
**Diastolic BP**	3.1 (0.5)*	0.4 (0.5)	0.8 (0.6)	-1.3 (0.6)*	2.3 (0.5)*	1.1 (0.7)*
**Total Cholesterol**	14.8 (1.4)*	12.2 (1.8)*	11.7 (2.3)*	14.5 (2.4)*	6.4 (1.9)*	6.5 (2.0)*
**HDL**	-7.2 (0.5)*	-8.7 (0.5)*	-4.3 (0.8)*	-6.9 (0.9)*	-3.2 (0.6)*	-5.7 (0.7)*
**HbA1C**	0.2 (0.03)*	0.1 (0.02)*	0.3 (0.04)*	0.2 (0.03)*	0.2 (0.04)*	0.1 (0.04)*
**Elevated CRP**	OR 5.2 (4.4–6.2)*	OR 3.7 (3.1–4.6)*	OR 7.5 (5.8–9.7)*	OR 5.4 (4.1–7.1)*	OR 5.2 (4.2–6.6)*	OR 3.8 (3.0–4.8)*
**Markers of kidney disease**						
**Albuminuria**	OR 1.2 (0.9–1.7)	OR 0.9 (0.6–1.3)	OR 1.4 (0.9–2.1)	OR 0.7 (0.4–1.4)	OR 3.5 (2.2–5.5)*	OR 3.0 (1.7–5.4)*
**Sex-specified Albuminuria**	OR 1.1 (0.8–1.5)	OR 0.9 (0.6–1.3)	OR 1.2 (0.9–1.6)	OR 0.8 (0.5–1.5)	OR 2.4 (1.7–3.5)*	OR 2.1 (1.4–3.2)*
**EGFR**	1.2 (0.7)	1.9 (0.6)*	4.9 (1.2)*	1.3 (1.3)	1.4 (0.7)*	-0.2 (0.7)

Fasting Sample Analysis	Non-Hispanic Whites (N = 1452) (38,957,077.4)		Non-Hispanic Blacks (N = 708) (7,351,235.5)		Mexican-Americans (N = 999) (10,353,432.3)	
	Unadjusted	Adjusted^¶^	Unadjusted	Adjusted^¶^	Adjusted	Unadjusted^¶^
**Triglycerides**	42.4 (3.2)*	9.0 (3.8)*	22.6 (5.5)*	10.2 (5.8)	29.5 (5.0)*	16.8 (4.7)*
**LDL**	10.2 (1.9)*	5.2 (2.5)*	10.9 (3.0)*	10.3 (3.2)*	3.3 (2.7)	3.5 (2.5)
**Glucose**	5.9 (1.0)*	2.0 (0.6)*	8.5 (2.2)*	0.9 (1.4)	1.5 (2.7)	-4.2 (1.1)*
**Insulin**	8.7 (0.7)*	5.9 (0.5)*	9.5 (0.6)*	6.8 (0.9)*	6.3 (0.9)*	4.8 (0.7)*
**HOMA**	1.2 (0.1)*	0.8 (0.1)*	1.1 (0.1)*	0.9 (0.1)*	0.9 (0.1)*	0.7 (0.1)*

^£^ For continuous variables: β (SE). For categorical variables: OR (95% C.I.)

* Statistically significant for pre-specified p<0.05

^¶^ Models adjusted for age, gender, income ratio, smoking status, survey year, and systolic blood pressure, total cholesterol, HDL-cholesterol, hemoglobin A1C, and C-reactive protein levels.

### CKD markers and abdominal obesity in normotensive, normoglycemic, normoinsulinemic individuals

A significant association of abdominal obesity with albuminuria persisted in Mexican-Americans [5.8 (1.7–20.2), p = 0.006] (Table 3). This association also remained significant in Mexican-Americans with normal insulin sensitivity [4.5 (1.6–12.2), p = 0.004](Table 3). Abdominal obesity remained significantly associated with a higher eGFR among non-Hispanic whites [β = 2.5 (1.2), p = 0.04], even in the model with insulin sensitivity [β = 3.4 (1.3), p = 0.01] (Table 3). In sensitivity analyses with normotensive blood pressures defined as ≤120/80mmHg, Mexican American young adults again showed significantly higher albuminuria with abdominal obesity, irrespective of insulin sensitivity, but not for sex-specified albuminuria (S4 Table). In the insulin sensitivity model, non-Hispanic whites showed a higher eGFR [β = 4.1 (1.4), p = 0.006], whereas blacks had significantly lower eGFR with abdominal obesity [β = -7.2 (3.3), p = 0.03] (S4 Table).

**Table 3:** Association of abdominal obesity with albuminuria among young adults 20–40 yrs with normal blood pressure and normoglycemia^£^.

Normotensive, normoglycemic	Non-Hispanic White (n = 967) (26,954,547)		Non-Hispanic Black (n = 454) (4,987,686.1)		Mexican-American (n = 526) (5,511,669.5)	
	Unadjusted	Adjusted^¶^	Unadjusted	Adjusted^¶^	Unadjusted	Adjusted^¶^
**Albuminuria**	OR 1.4 (0.7–2.7)	OR 1.4 (0.7–2.7)	OR 1.1 (0.4–2.8)	OR 0.8 (0.3–2.1)	OR 4.9 (1.7–14.4)*	OR 5.8 (1.7–20.2)*
**Sex-specified Albuminuria**	OR 1.3 (0.7–2.4)	OR 1.5 (0.8–2.9)	OR 0.7 (0.3–1.7)	OR 0.5 (0.2–1.3)	OR 2.6 (1.2–5.6)*	OR 3.6 (1.4–9.1)*
**eGFR**	1.3 (1.2)	2.5 (1.2)*	3.6 (2.3)	0.01 (2.7)	1.7 (1.5)	-1.5 (1.4)

Normotensive, normoglycemic, and without insulin resistance	Non-Hispanic White (n = 832) (23,497,353.4)		Non-Hispanic Black (n = 342) (3,739,268.9)		Mexican-American (n = 468) (4,954,341.1)	
	Unadjusted	Adjusted^¶^	Unadjusted	Adjusted^¶^	Unadjusted	Adjusted^¶^
**Albuminuria**	OR 1.3 (0.6–2.8)	OR 1.1 (0.4–2.9)	OR 0.5 (0.1–2.4)	OR 0.4 (0.1–1.2)	OR 5.4 (1.6–18.4)*	OR 4.5 (1.6–12.2)*
**Sex-specified Albuminuria**	OR 1.2 (0.6–2.3)	OR 1.2 (0.6–2.7)	OR 0.3 (0.1–1.4)	OR 0.2 (0.1–0.9)*	OR 3.4 (1.4–8.5)*	OR 2.6 (1.0–6.5)
**eGFR**	1.8(1.4)	3.4 (1.2)*	1.8 (3.0)	-2.1 (3.1)	1.5 (1.7)	-2.1 (1.6)

^£^ For continuous variables: β (SE). For categorical variables: OR (95% C.I.)

* Statistically significant for pre-specified p<0.05

^¶^ Models adjusted for age, gender, income ratio, smoking status, survey year, and systolic blood pressure, total cholesterol, HDL-cholesterol, hemoglobin A1C, and C-reactive protein levels.

### Physical activity sensitivity analyses

The level of physical activity did not alter the association of abdominal obesity with CKD risk factors or CKD markers in all adjusted analyses (data not shown).

### Kidney disease awareness

**Fig 1:**
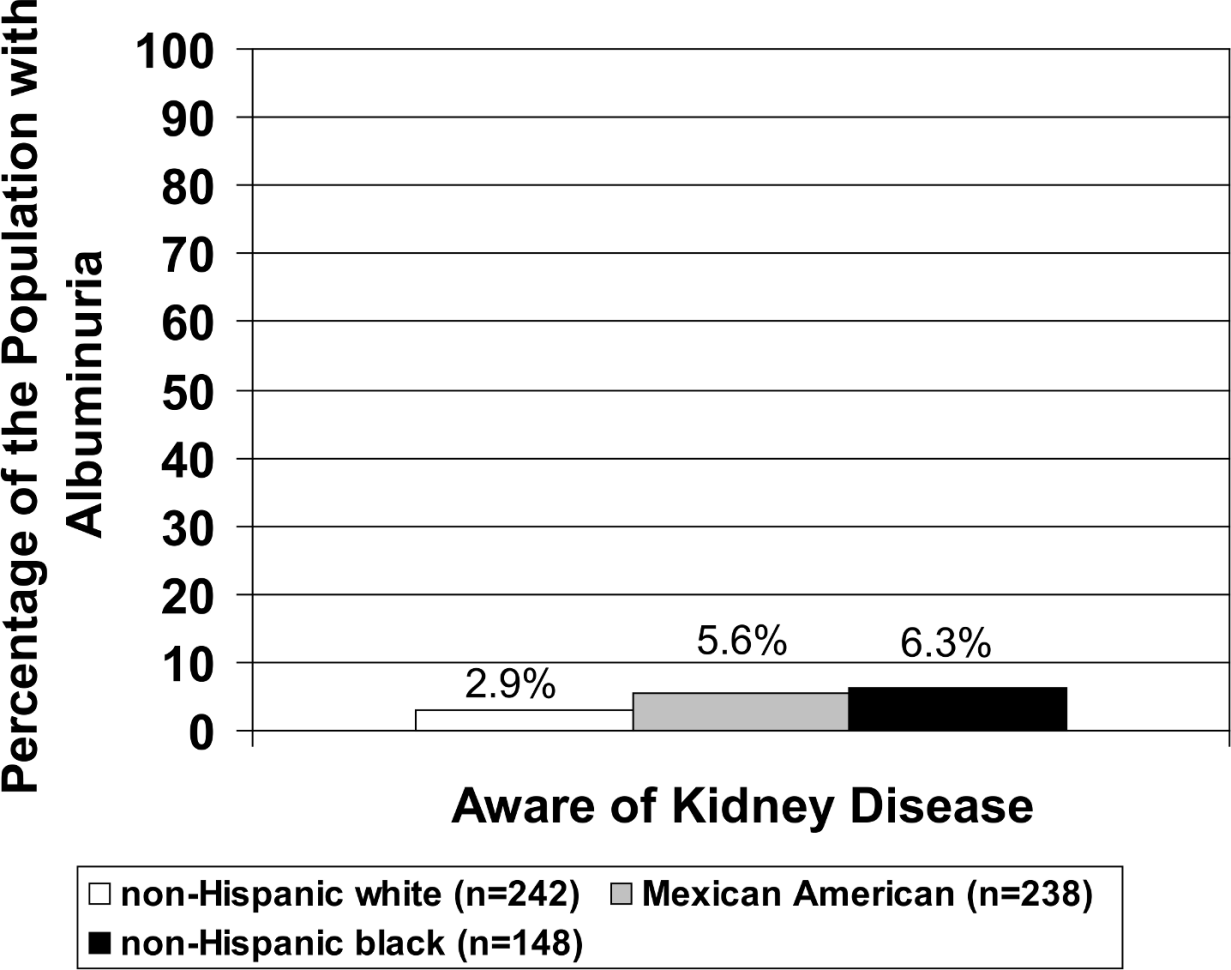
Prevalence of kidney awareness with race among young adults ages 20–40 years with albuminuria. Microalbuminuria defined as urine albumin: urine creatinine ratio ≥30 mg/g irrespective of gender.

Over 90% of young adults with albuminuria were unaware of kidney disease and this did not differ by race (p = 0.31). (Fig 1)

## Discussion

We assessed racial differences in the association of abdominal obesity with early CKD markers and CKD risk factors in young adults from a large nationally representative survey from 1999–2010, thereby representing recent experience in the US general population of young adults. We found a greater prevalence of abdominal obesity in non-Hispanic black and Mexican-American young adults than in non-Hispanic whites, similar to estimates in older adults [32]. We found significant associations between abdominal obesity and CKD risk factors and early CKD markers even in this young population. In addition, we found that non-Hispanic blacks had stronger associations between abdominal obesity and higher hemoglobin A1c and HOMA-IR levels, while non-Hispanic blacks and non-Hispanic whites showed stronger associations between abdominal obesity and lipid abnormalities compared to Mexican-Americans. CKD predominantly affects adults older than 40 years, and is unlikely to affect this young cohort [33]. However, we found a higher prevalence of early CKD markers in Mexican American young adults with abdominal obesity, suggesting that this fastest growing, but underserved, minority population in the United States may be more at risk for CKD when abdominal obesity is present and may need targeted screening and intervention.

Cardiovascular disease seems to be associated with markers of central adiposity more than with BMI [34–36]. As a surrogate for visceral fat, abdominal obesity possibly captures the effect of adipocyte-induced inflammation, better than BMI. Excess adiposity is associated with decreased renal blood flow, adipocyte-associated release of angiotensinogen and adipokines (e.g leptin), leading to a chronically activated renin-angiotensin system (RAS) [37, 38]. Visceral obesity mechanistically causes persistent efferent arteriolar vasoconstriction leading to glomerular hypertrophy and hyperfiltration in normal kidneys to maintain normal GFR [39, 40]. Albuminuria occurs later, secondary to sustained glomerular damage [39]. However, by the time albuminuria is apparent, progressive loss of glomerular function already seems to be occurring in diabetic and non-diabetic kidney disease, as shown by the PREVEND study and others [41–44].

We found evidence of albuminuria in Mexican American young adults with abdominal obesity, even after controlling for other CKD risk factors and markers of inflammation. More importantly, this finding persisted in healthy young Mexican Americans without hypertension, hyperglycemia or insulin resistance i.e., the most important risk factors for kidney disease, suggesting an independent direct effect of abdominal obesity. Albuminuria in obesity can be independent of diabetes mellitus, and improves with weight loss [28, 45]. Similar results were also seen in a cross-sectional study of apparently healthy Korean men (age 20–78 yrs, mean age 45yrs), also independent of insulin resistance [46].

The associations of abdominal obesity with eGFR were more variable and differed by race. The absolute differences in eGFR across races were small. However, these results suggest that there is a higher baseline eGFR in Mexican-Americans and non-Hispanic blacks than non-Hispanic whites, though it is not clear if this is because of glomerular hyperfiltration or a race-specific variation. It is also not clear if it is clinically significant. Though only non-Hispanic whites showed significant hyperfiltration with abdominal obesity in adjusted analyses, young non-Hispanic blacks and Mexican Americans may be already experiencing loss in glomerular function that is not evident statistically. Even within normal GFR ranges, obese black young adults demonstrated a faster GFR decline due to obesity, in the CARDIA Study [10]. Race also modifies the associations of abdominal obesity and other outcomes such as Mexican Americans being more susceptible to abdominal adiposity, metabolic syndrome, and the related non-alcoholic fatty liver disease (NAFLD) [47, 48]. These racial/ ethnic differences are important to elucidate in order to better target screening and treatment for those at highest risk.

Kidney disease remains asymptomatic until CKD stages 4 and 5 [49]. In a previous NHANES study (1988–1994 combined with 1999–2000), only about 10% of adults with moderate kidney disease (CKD stage III) were aware of their kidney disease, and greater awareness correlated with severity of renal dysfunction [50]. In our study, less than 5% of the young adults (20–40 years) with albuminuria had an awareness of their weak or failing kidneys. Albuminuria is associated with an increased cardiovascular risk even with normal kidney function in adults younger than 65 years of age [51].The duration of obesity appears to increase the risk of CKD, especially in individuals who are overweight or obese before 40 years [11]. This underlines the urgency of improving awareness of early kidney dysfunction in younger persons with abdominal obesity.

Our study has several limitations. The cross-sectional design of NHANES precludes assumptions of causality or temporality between abdominal obesity and CKD risk factors and early CKD markers. This also prevents ascertainment of persistent albuminuria for more than 3 months, as mandated to define CKD [28]. Measurement bias may result from the lack of clear, established cutoffs for hyperfiltration and an imprecise GFR estimating equation [26]. Creatinine-based measures, including UACR are influenced by age, gender and muscle mass. Blood pressure measurements were done on the same day and not assessed for variability with 24-hr ambulatory monitoring or multiple measurements on different days. Stratification compromised sample sizes. We could not account for residual confounders, including dietary and genetic differences across races. Lastly, our findings cannot be generalizable to other Hispanic groups. However, our study also has several important strengths such as a nationally representative sampling of the US population and systematic data collection.

## Conclusion

Abdominal obesity in young adults, especially in Mexican-Americans, is independently associated with early markers of kidney dysfunction even in individuals with normal blood pressures, glucose levels and insulin sensitivity. Abdominal obesity is also independently associated with other CVD risk factors with slight racial and ethnic differences in the associations. More clinical studies involving Mexican Americans, especially their genetics and environment, and the effect of weight loss interventions on kidney function are needed. Premature kidney dysfunction in abdominally obese young adults is likely to worsen with persistent obesity. Improving early recognition of kidney disease in younger adults at the patient and provider level is of public health importance.

## Supporting Information

S1 Table Weighted baseline characteristics of CKD risk factors and CKD markers that are categorical in distribution, with % reflecting row-wise proportions. (DOCX)

S2 Table Distribution of albuminuria by level among young adults 20–40 yrs. (DOCX)

S3 Table Weighted baseline characteristics of CKD risk factors and CKD markers in missing probands. (DOCX)

S4 Table Association of abdominal obesity with albuminuria among young adults 20–40 yrs with normal blood pressure (≤120/80mmHg) and normoglycemia (<100 mg/dl). (DOCX)
