# Supplementary material for: Abdominal Obesity, Race and Chronic Kidney Disease in Young Adults: Results from NHANES 1999-2010
Source: PLoS One. 2016 May 25;11(5):e0153588. doi: 10.1371/journal.pone.0153588 (PMC4880194; doi:10.1371/journal.pone.0153588)
Supplement: S1 Table — (DOCX) [file pone.0153588.s001.docx]

**Supplemental Table S1: Weighted baseline characteristics of CKD risk factors and CKD markers in participants with missing data.**

|  | **Non-Hispanic Whites**  **(N =413)** | | **Non-Hispanic Blacks**  **(N=354)** | | **Mexican-Americans**  **(N=405)** | |
| --- | --- | --- | --- | --- | --- | --- |
|  | **No abdominal obesity**  **278 (67.4%)** | **Abdominal Obesity**  **135 (32.5%)** | **No abdominal obesity**  **197 (54.2)** | **Abdominal Obesity**  **157 (45.8)** | **No abdominal**  **obesity**  **251 (62.3)** | **Abdominal Obesity**  **154 (37.7)** |
| **Age**(yrs)[Mean(SD)] | 28.8 | 30.7 | 30.2 | 31.8 | 28.2 | 29.5 |
| **Gender** [n (%)] | | | | | | |
| Males | 161 (57.8) | 55 (42.5) | 120 (58.7) | 52 (30.5) | 149 (59.9) | 54 (38.4) |
| Females | 117 (42.2) | 80 (57.5) | 77 (41.3) | 105 (69.5) | 102 (40.1) | 100 (61.6) |
| **Per-capita Income Ratio (PIR)** [n (%)] | | | | | | |
| Below poverty | 37 (17.4) | 21 (22.2) | 29 (24.3) | 35 (32.6) | 29 (29.8) | 24 (29.9) |
| Above poverty | 27 (15.2) | 16 (15.4) | 27 (21.3) | 31 (28.2) | 38 (31.4) | 30 (37.5) |
| 200% above poverty | 102 (67.4) | 48 (62.4) | 68 (54.4) | 45 (39.2) | 41 (38.8) | 24 (32.6) |
| **Smoking Status** [n (%)] | | | | | | |
| Never | 123 (44.9) | 69 (53.2) | 132 (67.7) | 101 (63.8) | 170 (68.4) | 97 (60.1) |
| Former | 41 (13.5) | 22 (15.3) | 9 (4.7) | 12 (8.6) | 18 (6.5) | 18(12.2) |
| Current | 116 (42.6) | 99 (31.4) | 55 (27.7) | 44 (27.6) | 62 (25.1) | 37 (27.7) |
| **BMI category** (kg/m^2^) [n (%)] | | | | | | |
| Normal (<25) | 193 (70) | 4 (2.3) | 112 (56.2) | 1 (0.8) | 142 (57.8) | 5 (2.1) |
| Overweight (25-29) | 78 (27.5) | 31 (24.3) | 77 (40.4) | 32 (20.1) | 97 (38.1) | 46 (24.6) |
| Obese (≥30) | 6 (2.5) | 99 (73.4) | 7 (3.4) | 121 (79.1) | 99(4.1) | 100 (73.2) |
| **BMI** (kg/m^2^) [Mean(SD)] | 23. 2 | 34.9 | 24.2 | 36.8 | 24.4 | 34.0 |
| **Systolic BP** (mmHg) **(**Mean(SD)] | 113.0 | 119.3 | 119.1 | 120.8 | 114.0 | 117.2 |
| **Diastolic BP** (mmHg)[Mean(SD)] | 67.3 | 70.9 | 72.5 | 74.2 | 67.0 | 71.9 |
| **Hypertension** [n (%)] | 14 (6.4) | 23 (20.7) | 19 (12.6) | 27 (26.7) | 6 (3.0) | 10 (12.2) |
| **HbA1C** (%)[Mean(SD)] | 5.1 | 5.3 | 5.2 | 5.9 | 5.2 | 5.8 |
| **Total Cholesterol** (mg/dl) [Mean(SD)] | 186.6 | 197.1 | 173.6 | 188.2 | 187.6 | 193.6 |
| **HDL-Cholesterol** (mg/dl) [Mean(SD)] | 55.2 | 42.5 | 57.9 | 49.1 | 54.0 | 44.9 |
| **Elevated CRP** (>21mg/dl) [n (%)] | 140 (50.5) | 110 (80.8) | 113 (59.8) | 132 (84.2) | 111 (39.7) | 123 (78.2) |
| **Markers of kidney damage** | | | | | | |
| **eGFR**[Mean (SD)] (ml/min/1.73m^2^) | 106.8 | 105.3 | 114.1 | 117.9 | 117.2 | 111.3 |
| **Albuminuria** (mg/g) [n(%)] | 24 (9.5) | 22 (16.0) | 15 (7.8) | 34 (21.6) | 30 (10.9) | 26 (18.6) |
| **Sex-specific albuminuria** (mg/g) [n(%)] | 23 (9.7) | 19 (14.1) | 21 (10.8) | 31 (19.6) | 28 (10.2) | 23 (17.5) |
|  | **Non-Hispanic Whites**  **(N =149)** | | **Non-Hispanic Blacks**  **(N=217)** | | **Mexican-Americans**  **(N=236)** | |
|  | **No abdominal obesity**  **170 (64.7 %)** | **Abdominal Obesity**  **86 (35.5 %)** | **No abdominal obesity**  **125 (56.9 %)** | **Abdominal Obesity**  **92 (43.1 %)** | **No abdominal**  **obesity**  **152 (68.7 %)** | **Abdominal Obesity**  **84 (31.3)** |
| **Triglycerides** (mg/dl) [Mean(SD)] | 202.1 | 287.8 | 103.8 | 198.6 | 164.6 | 232.3 |
| **LDL-Cholesterol** (mg/dl) [Mean(SD)] | 111.9 | 122.2 | 99.2 | 116.1 | 107.2 | 117.7 |
| **Glucose** (mg/dl) [Mean(SD)] | 92.7 | 100.6 | 91.0 | 118.1 | 94.8 | 123.5 |
| **Insulin** (mg/dl) [Mean(SD)] | 7.1 | 19.6 | 6.8 | 19.8 | 9.0 | 15.6 |
| **HOMA** [Mean (SD)] | 2.0 | 3.3 | 1.7 | 3.3 | 2.0 | 3.3 |
| **Diabetes status [n (%)]** |  |  |  |  |  |  |
| Non-diabetic | 104 (82.2) | 40 (60.7) | 74 (90.6) | 40 (62.1) | 102 (79.4) | 35 (53.0) |
| Glucose intolerance | 19 (15.3) | 22 (32.6) | 13 (9.4) | 10 (22.5) | 29 (18.9) | 22 (31.2) |
| Diabetes | 4 (2.5) | 6 (6.7) | 1 (0) | 9 (15.4) | 2 (1.7) | 9 (15.7) |
| **Insulin resistance status [n (%)]** |  |  |  |  |  |  |
| Normal | 76 (91.0) | 24 (50.3) | 50 (96.0) | 19 (49.5) | 87 (90.0) | 28 (54.3) |
| Insulin resistance (HOMA ≥75^th^%tile) | 8 (9.1) | 28 (49.7) | 2 (4.0) | 20 (50.5) | 11 (10.0) | 24 (45.7) |

^¥^ For categorical variables, % are calculated from column-wise proportion

*Standard errors could not be determined.
