# Supplementary material for: Abdominal Obesity, Race and Chronic Kidney Disease in Young Adults: Results from NHANES 1999-2010
Source: PLoS One. 2016 May 25;11(5):e0153588. doi: 10.1371/journal.pone.0153588 (PMC4880194; doi:10.1371/journal.pone.0153588)
Supplement: S2 Table — (DOCX) [file pone.0153588.s002.docx]

**Supplemental Table S2: Weighted baseline characteristics of CKD risk factors and CKD markers that are categorical in distribution, with % reflecting row-wise proportions**

|  | | **Non-Hispanic Whites**  **(N =3226) (37,606,080.5)** | | | | | | **Non-Hispanic Blacks**  **(N=1440) (6,579,829.4)** | | | | | | **Mexican-Americans**  **(N= 2252) (10,111,528.9)** | | | | |
| --- | --- | --- | --- | --- | --- | --- | --- | --- | --- | --- | --- | --- | --- | --- | --- | --- | --- | --- |
|  | | **No abdominal obesity**  **1897(62.8%)** | **Abdominal Obesity**  **1329 37.2%)** | | **P** | | | **No abdominal obesity**  **781 (54.8%)** | | **Abdominal Obesity**  **659 (45.2%)** | | **p** | | **No abdominal obesity**  **1244(59.6%)** | | **Abdominal Obesity**  **1008 (40.4%)** | | **P** |
| **Poverty Income Ratio (PIR)** [n(%)] | | | | | | | | | | | | | | | | | | |
| Below poverty | | 259 (60.6) | 218 (39.4) | | 0.37 | | | 205 (53.5) | | 189 (46.5) | | 0.39 | | 377 (55.8) | | 341 (44.2) | | 0.25 |
| Above poverty | | 367 (60.6) | 259 (39.4) | |  |  |  | 195 (52.0) | | 180 (48.0) | |  |  | 442 (61.2) | | 341 (38.8) | |  |
| 200% above poverty | | 1271 (63.5) | 852 (36.5) | |  |  |  | 381 (56.8) | | 290 (43.2) | |  |  | 425 (60.7) | | 326 (39.3) | |  |
| **Smoking Status** [n (%)] | | | | | | | | | | | | | | | | | | |
| Never | | 939 (63.1) | 663 (36.9) | | 0.03 | | | 519 (53.0) | | 466 (47.0) | | 0.003 | | 780 (57.8) | | 700(42.2) | | 0.04 |
| Former | | 280 (57.0) | 264 (43.0) | |  |  |  | 399 (44.5) | | 55 (55.5) | |  |  | 169 (57.6) | | 149 (42.4) | |  |
| Current | | 678 (64.6) | 402 (35.4) | |  |  |  | 223(61.5) | | 138 (38.5) | |  |  | 295 (65.0) | | 159 (35.0) | |  |
| **Fasting Sample Analysis** | | **Non-Hispanic Whites**  **(N =1452) (38,957,077.4)** | | | | | | **Non-Hispanic Blacks**  **(N= 708) (7,351,235.5)** | | | | | | **Mexican-Americans**  **(N= 999) (10,353,432.3)** | | | | |
|  | | **No abdominal obesity**  **857 (63.2)** | **Abdominal Obesity**  **595 (36.8)** | | **p** | | | **No abdominal obesity**  **386 (53.6)** | | **Abdominal Obesity**  **322 (46.3)** | | **p** | | **No abdominal obesity**  **536 (56.8)** | | **Abdominal Obesity**  **463 (43.2)** | | **p** |
| **Diabetes status [n (%)]** | | | | | | | | | | | | | | | | | | |
| Non-diabetic | 687 (67.6) | | 425 (32.4) | | | <0.001 | | 319 (58.0) | | 222 (42.0) | | <0.001 | | 359 (55.8) | | 318 (44.2) | | 0.19 |
| Glucose intolerance | 165 (51.6) | | 152 (48.4) | | |  |  | 67 (44.0) | | 86 (56.0) | |  |  | 170 (60.3) | | 146 (39.7) | |  |
| Diabetes | 5 (24.9) | | 18 (75.1) | | |  |  | 0 (0) | | 14 (100.0) | |  |  | 7 (34.9) | | 9 (65.1) | |  |
| **Insulin resistance status [n (%)]** | | | | | | | | | | | | | | | | | | |
| Normal | 760 (74.1) | | | 324 (25.9) | | | <0.001 | | 310 (69.9) | | 135 (30.1) | | <0.001 | | 412 (81.9) | | 231 (52.2) | <0.001 |
| Insulin resistance (HOMA ≥75^th^%tile) | 59 (21.8) | | | 237 (78.2) | | |  |  | 49 (21.6) | | 169 (78.4) | |  |  | 98 (18.1) | | 216 (47.8) |  |

^¥^ For categorical variables, % are calculated from column-wise proportions.
