# Supplementary material for: Abdominal Obesity, Race and Chronic Kidney Disease in Young Adults: Results from NHANES 1999-2010
Source: PLoS One. 2016 May 25;11(5):e0153588. doi: 10.1371/journal.pone.0153588 (PMC4880194; doi:10.1371/journal.pone.0153588)
Supplement: S3 Table — (DOCX) [file pone.0153588.s003.docx]

**Supplemental Table 3: Distribution of albuminuria by level among young adults 20-40 yrs**

|  | **Non-Hispanic Whites**  **(N =3226) (37,606,080.5)** | | | **Non-Hispanic Blacks**  **(N=1440) (6,579,829.4)** | | | **Mexican-Americans**  **(N= 2252) (10,111,528.9)** | | |
| --- | --- | --- | --- | --- | --- | --- | --- | --- | --- |
| **Albuminuria** | **No abdominal obesity**  **1897(62.8%)** | **Abdominal Obesity**  **1329 37.2%)** | **P** | **No abdominal obesity**  **781 (54.8%)** | **Abdominal Obesity**  **659 (45.2%)** | **p** | **No abdominal obesity**  **1244(59.6%)** | **Abdominal Obesity**  **1008 (40.4%)** | **P** |
| **Albuminuria (UACR≥30mg/g)**  **[n (%)]** | 88 (4.5) | 77 (5.6) | 0.17 | 37 (4.8) | 46 (6.6) | 0.09 | 48 (3.6) | 101 (11.6) | <0.001 |
| UACR 30-100mg/g [n(%)] | 72 (3.7) | 56 (4.4) | 0.12 | 21 (2.8) | 31 (4.4) | 0.44 | 29 (2.2) | 70 (8.6) | <0.001 |
| UACR 100-300mg/g [n(%)] | 10 (0.5) | 18 (1.1) |  | 7 (0.9) | 8 (1.0) |  | 10 (0.8) | 18 (1.9) |  |
| UACR>300mg/g [n(%)] | 6 (0.3) | 3 (0.1) |  | 9 (1.1) | 7 (1.2) |  | 9 (0.6) | 13 (1.2) |  |
