# Supplementary material for: Abdominal Obesity, Race and Chronic Kidney Disease in Young Adults: Results from NHANES 1999-2010
Source: PLoS One. 2016 May 25;11(5):e0153588. doi: 10.1371/journal.pone.0153588 (PMC4880194; doi:10.1371/journal.pone.0153588)
Supplement: S4 Table — (DOCX) [file pone.0153588.s004.docx]

**Supplemental Table 4: Association of abdominal obesity with albuminuria among young adults 20-40 yrs with normal blood pressure (≤120/80mmHg) and normoglycemia (<100 mg/dl)**^£^

| **Normotensive, normoglycemic** | **Non-Hispanic White**  **(n=814) (22,298,519.5)** | | **Non-Hispanic Black**  **(n=318) (3,486,279.5)** | | | **Mexican-American**  **(n=497) (5,097,145.1)** | | | |
| --- | --- | --- | --- | --- | --- | --- | --- | --- | --- |
|  | **Unadjusted** | **Adjusted**^¶^ | **Unadjusted** | **Adjusted**^¶^ | | **Unadjusted** | | **Adjusted**^¶^ | |
| **Albuminuria** | OR 1.2 (0.6-2.2) | OR 1.4(0.6-3.0) | OR 1.3(0.4-4.1) | OR 0.8 (0.3-2.1) | | OR 5.3 (1.8-15.8)* | | OR 4.0 (1.5-10.7)* | |
| **Sex-specified Albuminuria** | OR 1.0 (0.6-1.9) | OR 1.2 (0.6-2.4) | OR 1.1(0.5-2.6) | OR 0.6(0.2-1.6) | | OR 2.5 (1.1-5.6)* | | OR 2.0 (0.9-4.5) | |
| **eGFR** | 1.8 (1.3) | 2.8 (1.4)* | 1.6 (2.4) | -3.3 (2.9) | | 1.7 (2.4) | | -1.0 (1.9) | |
|  | | | | | | | | | |
| **Normotensive, normoglycemic, and normal insulin levels** | **Non-Hispanic White**  **(n=685) (18,993,873.4)** | | **Non-Hispanic Black**  **(n=239) (2,625,660.5)** | | | | **Mexican-American**  **(n=396) (4,125,678.4)** | | |
|  | **Unadjusted** | **Adjusted**^¶^ | **Unadjusted** | | **Adjusted**^¶^ | | **Unadjusted** | | **Adjusted**^¶^ |
| **Albuminuria** | OR 1.0(0.4-2.4) | OR 1.0 (0.3-3.1) | OR 1.0 (0.2-4.2) | | OR 1.1 (0.3-4.3) | | OR 6.6 (2.0-21.9)* | | OR 3.9(1.4-10.9)* |
| **Sex-specified Albuminuria** | OR 0.8 (0.4-1.9) | OR 0.9(0.3-2.4) | OR 0.6 (0.2-2.5) | | OR 0.3 (0.1-1.6) | | OR 3.7(1.5-9.1)* | | OR 2.1 (0.9-4.7) |
| **eGFR** | 2.8 (1.5) | 4.1 (1.4)* | -0.2 (3.0) | | -7.2 (3.3)* | | 2.3 (2.7) | | -0.7 (2.3) |

^£^For continuous variables : β (SE). For categorical variables: OR (95% C.I.)

^*^Statistically significant for pre-specified p<0.05

^¶^Models adjusted for age, gender, income ratio, smoking status, survey year, and systolic blood pressure, total cholesterol, HDL-cholesterol, hemoglobin A1C, and C-reactive protein levels.
